# Blood volume-sensitive laminar fMRI with VASO in human hippocampus: Capabilities and biophysical challenges at clinical 7T scanners

**DOI:** 10.1162/IMAG.a.1197

**Published:** 2026-04-09

**Authors:** Khazar Ahmadi, Stephanie Swegle, Sriranga Kashyap, Antoine Bouyeure, Peter Bandettini, Nikolai Axmacher, Laurentius Huber

**Affiliations:** Department of Neuropsychology, Institute of Cognitive Neuroscience, Faculty of Psychology, Ruhr University Bochum, Bochum, Germany; National Institutes of Health, Bethesda, MD, United States; Krembil Brain Institute, University Health Network, Toronto, ON, Canada; Martinos Center, MGH, Harvard Medical School, Charlestown, MA, United States

**Keywords:** laminar fMRI, VASO contrast, hippocampus, memory, ultra-high field

## Abstract

Sub-millimeter resolution functional magnetic resonance imaging (fMRI) at ultra-high field (≥ 7T) has offered an unprecedented opportunity to probe mesoscopic computations at a columnar or laminar level. However, its application has been primarily restricted to the neocortex. Inferior brain regions, particularly the hippocampus (HC), are challenging targets for laminar fMRI. Recent developments in acquisition methods have shown the feasibility of laminar recordings in the HC using gradient-echo blood oxygenation level-dependent (BOLD) contrast. Nonetheless, the spatial specificity of the BOLD signal is compromised by the draining veins’ bias. Cerebral blood volume (CBV)-sensitive sequences including vascular space occupancy (VASO) have emerged as a promising approach to capture the laminar activity with mitigated venous bias. Yet, its feasibility in the HC is unclear and challenged by methodological constraints. Here, we optimized VASO to mitigate the macrovasculature contribution in HC. By evaluating a series of advanced acquisition strategies tailored to HC, we obtained improved VASO signal quality with minimal artifacts. The optimized protocol was further validated with an autobiographical memory task. Our findings show that combining the high detection power of gradient-echo BOLD with the vein-bias-mitigated VASO contrast allows for differentiation between neural activity-related BOLD signals and those biased by draining veins. These results demonstrate the feasibility of submillimeter VASO acquired with conventional 7T scanners in the HC to map the circuit-level mechanisms of memory retrieval across HC subfields, laying a foundation to investigate the microcircuitry of HC-driven complex cognitive functions and their alterations in neurodegeneration and epilepsy.

## 1 Introduction

Laminar fMRI is a rapidly growing field that enables the dissociation of feedforward and feedback projections, each targeting distinct cortical layers (Stephan et al., 2019). This offers an opportunity to examine the mesoscopic organization of the brain at higher precision, facilitating the integration of microscopic insights from animal and postmortem studies with macroscopic findings from behavioral and conventional neuroimaging research in humans (Chai & Zhang, 2024; Lawrence et al., 2019; Stephan et al., 2019; Yang et al., 2021). Despite its increasing popularity, the vast majority of laminar fMRI studies have focused on the neocortex (De Martino et al., 2015; Finn et al., 2019; Huber et al., 2017; Kok et al., 2016; Muckli et al., 2015; Olman et al., 2012; Sharoh et al., 2019) while the underlying microcircuitry of allocortex, especially the hippocampus (HC), has been sparsely studied using this approach (Maass et al., 2014; Pfaffenrot et al., 2025).

**Fig. 1.**
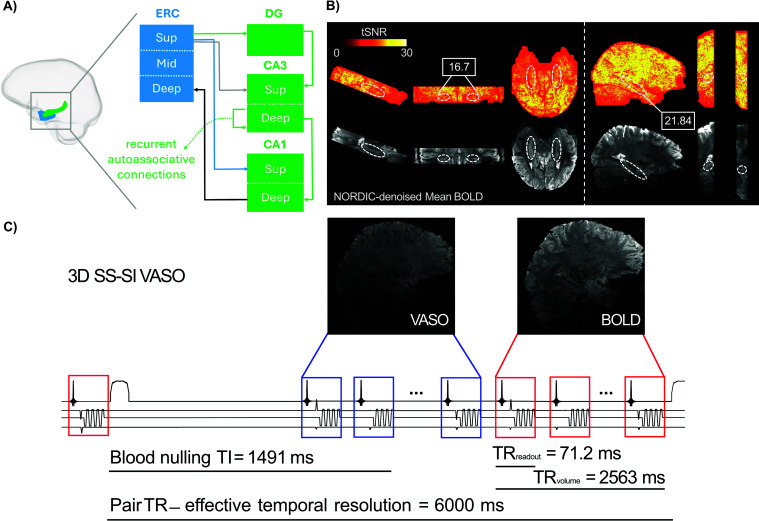
Overview of hippocampal projections and VASO optimization procedure. (A) Schematic illustration of HC-ERC microcircuitry. The trisynaptic pathway (green) includes projections between ERC, DG, CA3, and CA1, while the monosynaptic (temporoammonic) pathway (blue) comprises direct connections from ERC to CA1. There are also direct connections between ERC and CA3, depicted in gray. The HC output (black) targets deep layers of ERC. The dashed green arrow represents autoassociative connections between CA3 neurons. ERC = entorhinal cortex, HC = hippocampus, Sup and Mid = superficial and middle layers, respectively. The SRLM (not shown here) lies within the superficial compartment in CA1. (B) tSNR maps and mean BOLD images from one participant, acquired using the reference 3D BOLD-based EPI sequence (left panel) with bilateral HC coverage and imaging slab oriented parallel to the HC long axis. The right panel shows the resulting images after adjustment of multiple parameters including FOV reduction, targeting left HC in sagittal orientation in the same participant. The optimization led to improved tSNR, as shown in the white box. This improvement is expected to generalize across participants since the adjustments systematically reduce artifacts caused by scanner hardware-induced EPI phase inconsistencies. Dashed circular markers indicate approximate location of HC. (C) Schematic depiction of HC-tailored 3D SS-SI VASO sequence protocol that was developed by further refining the acquisition parameters in (B) right panel. Note that ~ 500 ms was dedicated to slice-wise fat suppression modules, increasing the ultimate volume TR to 3000 ms and effective temporal resolution to 6000 ms. TR = repetition time, TI = inversion time, SS-SI = slice-selective slab inversion.

The HC, situated at the apex of the ventral visual stream, plays a key role in complex cognitive functions including memory and navigation. Although these functions have been well characterized at the macroscale, predominantly using conventional fMRI with spatial resolution of approximately 2 to 3 mm (Cohn-Sheehy et al., 2021; Ekstrom et al., 2003; Kim, 2011), their circuit-level substrates are poorly understood. This is partially due to signal drop-out associated with its proximity to air cavities that distort the magnetic field (Koster et al., 2018; Maass et al., 2014; Pfaffenrot et al., 2025). Furthermore, high-resolution fMRI of the HC is constrained by its large distance to radiofrequency (RF) coil elements, the large matrix sizes required, and the relatively short T2* values. These factors make it difficult to achieve the desired short echo times (TE) with minimal undersampling, limit attainable signal-to-noise ratios (SNR), and ultimately complicate imaging of the HC at ultra-high field (Huber, Stirnberg, et al., 2025). Additionally, the cytoarchitectonic laminar structure of the HC and its macrovasculature differ markedly from those of the neocortex (Duvernoy, 2005), posing additional hurdles for the analysis of laminar fMRI data in this subcortical region. Unlike the neocortex with a canonical six-layered structure, the HC exhibits a three-layered laminar architecture arranged in a radial fashion following its curvature, similar to layers of an onion (Pfaffenrot et al., 2025). Importantly, HC is not a homogenous structure, rather it comprises multiple subfields including subiculum, dentate gyrus (DG), and four cornu ammonis areas (CA1 to CA4), with CA4 also referred to as the hilus of the DG (Amaral, 1978; Insausti & Amaral, 2004). These subfields are interconnected with neocortical areas via the entorhinal cortex (ERC). This is mediated by two major, anatomically distinct circuits (Fig. 1A), the trisynaptic pathway that links superficial layers of ERC to DG and then to proximal apical dendrites of CA3 and subsequently to CA1 via the Schaffer collaterals; and the monosynaptic (temporoammonic) pathway that directly couples ERC to superficial layers of CA1 at distal apical dendrites in stratum radiatum lacunosum moleculare (SRLM). Within CA3, pyramidal neurons are interconnected through a recurrent autoassociative network toward stratum radiatum (Le Duigou et al., 2014; Witter, 2007). In contrast, the HC output originates from deep layers of CA1 at stratum pyramidale and stratum oriens and is later projected to deep layers of ERC (Koster et al., 2018; Maass et al., 2014; Rolls, 2013; Turk-Browne, 2019).

Despite these challenges, recent advancements in gradient-echo sequences with blood oxygenation level-dependent (GE-BOLD) contrast and echo planar imaging (EPI) readouts (Stirnberg & Stöcker, 2021) alongside the availability of new analysis approaches and software packages have driven an increasing interest in mapping the laminar organization of HC (Ahmadi et al., 2024; Pfaffenrot et al., 2025). While the BOLD contrast provides a relatively high SNR (Dumoulin et al., 2018; Yacoub et al., 2005), it is prone to venous drainage effects. This is particularly pertinent in HC where the blood can drain from either the inner surface (i.e., superficial layers) or outer surface (i.e., deep layers; see Duvernoy, 2005). The contribution of large vessels to laminar BOLD responses has been extensively studied in the neocortex and several physiological models have been developed to account for venous bias (Báez-Yáñez et al., 2025; Havlicek & Uludağ, 2020; Markuerkiaga et al., 2016). However, such models are currently lacking for the HC whose vasculature is substantially different from the neocortex. This warrants the application of alternative acquisition approaches such as blood volume-weighted laminar fMRI with vascular space occupancy (VASO) that hold a great potential to augment the high sensitivity of BOLD with an additional measure of vein-bias-mitigated laminar-specific activation (Huber et al., 2014). VASO has been successfully implemented to resolve laminar activity across various cortical areas (Faes et al., 2023; Finn et al., 2019; Huber et al., 2023; Pizzuti et al., 2023). Nonetheless, its application in HC remains unexplored. Building on a previous laminar fMRI study with BOLD contrast (Pfaffenrot et al., 2025), we sought to develop an effective VASO protocol to map the laminar organization of HC across typical 7T scanners. Here, we first outline a series of advanced acquisition strategies to mitigate the physiological and methodological challenges of VASO in the HC that are essential for optimizing an HC-tailored imaging protocol. We then present the results of a validation study to assess the laminar profiles of the HC subfields during an autobiographical memory paradigm (Leelaarporn et al., 2024; McCormick et al., 2015; Pfaffenrot et al., 2025). Our findings establish the feasibility of mesoscale VASO recordings in HC, providing groundwork for future studies to decipher the circuit-level mechanisms of high-level cognitive processes in HC.

## 2 Methods

### 2.1 Participants

Thirteen healthy volunteers (6 females, mean age = 30.7 years) were recruited from the National Institutes of Health (NIH) community after providing written informed consent according to procedures approved by the NIH Institutional Review Board. A total of 18 scanning sessions were conducted where 10 participants each completed a single session, while 3 participants took part in multiple sessions (2 participants completed 2 sessions each, and 1 participant completed 4 sessions). The study was conducted in two parts. The first part focused on optimizing the VASO sequence, during which three participants underwent four sessions. The second part, aimed at validating the optimized sequence with an autobiographical memory paradigm, involved 11 participants across 14 sessions. Data from five individuals were excluded from the second part due to experimental errors (stimulus onset not synchronized with the sequence trigger, N = 2) or excessive motion artifacts (N = 3), leaving six usable datasets acquired over nine sessions. Although no statistical tests were used to pre-determine the required sample size, the number of recruited individuals aligns with a previous BOLD-based laminar fMRI study utilizing the same task (Pfaffenrot et al., 2025). Nevertheless, given the resulting sample size (N = 6), the validation analysis should be interpreted as a feasibility-first demonstration rather than a fully powered evaluation of the paradigm.

### 2.2 Part 1: Developing an HC-tailored VASO protocol

Three 7T SIEMENS scanners, two identical MAGNETOM TERRA and a classic 7T Plus systems (SIEMENS Healthineers, Erlangen, Germany), were used depending on the availability of free timeslots. Each scanner was equipped with a 32 receive channel head coil (NOVA Medical, Wilmington, MA). All scanners operated with the same sequence version of the same software baseline VE12U, utilizing the same gradient strengths. Third-order shims were unplugged to reduce gradient resonance artifacts known as Fuzzy Ripples that are associated with magneto-mechanical coupling between third-order shims and read-out gradients (Huber, Stirnberg, et al., 2025). Additionally, the parallel transmission (pTX) system was used because of its larger transmit coverage in lower brain areas (Huber, Stirnberg, et al., 2025). To quantify temporal signal-to-noise ratio (tSNR) in the HC, we first employed a reference 3D GE-EPI sequence with BOLD contrast (Stirnberg & Stöcker, 2021) in line with previous studies (Ahmadi et al., 2024; Pfaffenrot et al., 2025). Briefly, the key parameters were TR|TE = 2500|28.4 ms, voxel size = 0.8 x 0.8 x 0.8 mm^3^, FOV = 192 mm, GRAPPA = 4, base resolution = 240, FA = 14°, bandwidth = 1096 Hz/px, partial Fourier = 7/8, phase-encoding direction = AP and 40 slices where imaging slab covered bilateral hippocampi and was placed parallel to their long axis. To boost the tSNR, the imaging slab was positioned in sagittal orientation with unilateral HC coverage (see Fig. 1B) and several acquisition parameters of the 3D GE-EPI sequence (Stirnberg & Stöcker, 2021) were adjusted as following: FOV = 180 mm, GRAPPA = 3, base resolution = 226, partial Fourier = 6/8, bandwidth = 1106 Hz/px, TR|TE = 2664|21.8 ms, FA = 18°. Leveraging this optimized acquisition setup, a 3D slice-selective slab-inversion (SS-SI) VASO sequence was implemented to acquire concomitant VASO and BOLD images in an interleaved fashion (Huber et al., 2014). VASO uses an inversion recovery pulse to null the blood signal enabling the measurement of cerebral blood volume changes through the residual tissue signal (Hua et al., 2013; Lu et al., 2003). A major methodological challenge of VASO in the HC is the relatively short arterial arrival time (Rane et al., 2016) that can lead to inflow of fresh (non-inverted) blood during VASO read-out, manifesting as bright spots in the acquired data. To implement a VASO protocol tailored to HC, we adjusted a wide range of parameters including read-out and volume TR, TE, FA, TI1, TI2, inversion delay, RF power scale, and RF bandwidth time product (BWTP). The optimum set of parameters were determined with respect to tSNR and minimal inflow contamination in HC (see Sections 2.3 and 3). Following the optimization of the VASO sequence, one participant was re-scanned with a single transmit (1sTX/32Rx) head coil allowing for direct comparison with pTX coil. For anatomical reference, whole-brain T1-weighted images were acquired using 3D Magnetization Prepared 2 Rapid Acquisition Gradient Echo (MP2RAGE) sequence (Marques et al., 2010) at an isotropic resolution of 0.75 mm^3^ with TR|TE = 4550|1.94 ms, TI1|TI2 = 840|2370 ms, FA1|FA2 = 5°|6°, and matrix size = 240 x 320 x 320.

### 2.3 Part 2: Sequence validation with an autobiographical memory paradigm

#### 2.3.1 Experimental task

The autobiographical memory paradigm was implemented in a block design using PsychoPy (Peirce et al., 2019), adapted from previous studies (Leelaarporn et al., 2024; McCormick et al., 2015; Pfaffenrot et al., 2025). It consisted of 2 conditions: (i) autobiographical memory retrieval and (ii) math with 15 trials per condition. The math trials served as a baseline since mental arithmetic primarily activates the parietal cortex (Dehaene et al., 2005; Istomina & Arsalidou, 2024; Rueckert et al., 1996) and does not rely on episodic memory and HC function (Leelaarporn et al., 2024). Each trial lasted for 18 seconds followed by an inter-trial interval of 12 seconds during which a central blue square was displayed on a white background. During memory trials, participants were presented with general word cues, for example, “beach.” They were instructed to recall a cue-related event from their personal past and re-experience it as vividly as possible for the remainder of the trial. To ensure the HC involvement, participants were required to retrieve recent memories (no older than 2 to 3 years) as remote episodic memories may be more strongly represented in neocortical regions (Bonnici et al., 2012; Takashima et al., 2009). In the math condition, participants solved a simple addition or subtraction problem, for example, “74 – 35”. Upon finding a solution, they were instructed to iteratively add 3 to their answer e.g., (39 + 3 + 3 + 3 …) until the end of the trial. Prior to the scanning, participants were thoroughly briefed on the experimental procedure. Each participant completed a minimum of 3 runs, with each run consisting of 15 trials featuring a unique set of word cues and math operations. The stimuli were presented on a rear-projection screen located at the magnet bore and viewed through an angled mirror.

#### 2.3.2 Data acquisition

Using the optimized protocol, we acquired concurrent VASO and BOLD data from six participants across nine sessions with the same scanners described in part 1. Each session consisted of three runs. Toward the end of each session, participants were asked via intercom whether they were willing to complete an optional fourth run. As a result, four runs were obtained in three participants. A few runs in some participants were discarded due to excessive motion artifacts, thresholded as maximum displacement of 2.5 mm due to rotation or translation. Detailed information on the number of acquired sessions and analyzed datasets per participant is provided in Supplementary Table S1 while Supplementary Table S2 summarizes the estimated absolute and relative motion for each run in every participant. The acquisition parameters were as follows: Inversion-recovery 3D-EPI with isotropic voxel size of 0.8 mm^3^, 36 slices, FOV = 180 mm, read-out TR | volume TR | TE = 71.2, 6000, 23.90 ms, PE-direction = AP, variable flip angles with reference (last) nominal value of FA = 35°, base resolution = 226, bandwidth = 1106 Hz/pixel, GRAPPA = 3, partial Fourier = 6/8, RF BWTP = 12, RF power scale = 3, TI1 = 1491 ms. Each functional run comprised 308 time points, corresponding to 154 pairs of interleaved VASO and BOLD volumes with a total duration of 15.4 minutes. In addition, anatomical data were acquired in all participants with identical parameters specified in part 1. When possible (N = 5 out of 6), an auxiliary fMRI scan with opposite phase-encoding direction, that is, PA, was acquired to facilitate correction of susceptibility-induced distortion artifacts.

### 2.4 Data analysis

#### 2.4.1 Anatomical data processing

The unified image (UNI) of the MP2RAGE sequence was first preprocessed using Presurfer (Kashyap, 2021) to remove background noise by utilizing a bias-corrected image corresponding to second inversion time (INV2). The resulting denoised image was then inputted to HippUnfold package (DeKraker et al., 2022), executed via Singularity, to automatically segment HC subfields and delineate its surface boundaries (Supplementary Fig. S1). Although T2-weighted structural images are generally favored for HC segmentation owing to superior contrast between HC gray matter (GM) and the SRLM tissue, recent advances in HippUnfold enable robust and accurate HC segmentation on T1-weighted data (DeKraker et al., 2022; Haast et al., 2024). The denoised T1 image was further skull-stripped using FSL brain extraction tool (BET; see Smith, 2002) and subsequently segmented with FSL FAST (Zhang et al., 2002) to classify three tissue types corresponding to GM, white matter (WM), and cerebrospinal fluid (CSF).

#### 2.4.2 Functional data preprocessing

All functional runs were sorted by contrast yielding separate VASO and BOLD time series, each comprising 154 volumes (Moia et al., 2024). The first two volumes of each contrast were discarded in every run to allow magnetization to reach a steady state. Of the 36 acquired slices, 3 were removed from the outermost edge of the acquisition slab due to distortion artifacts. Noise Reduction with Distribution Corrected Principle Component Analysis (NORDIC-PCA; see Knudsen et al., 2025; Moeller et al., 2021; Vizioli et al., 2021) was applied for each contrast separately to boost the inherently low SNR of the laminar fMRI data through thermal noise suppression. The last 2 volumes, which are typically used for estimation of the noise threshold in NORDIC, were also excluded resulting in a total of 150 volumes per contrast. Motion and distortion correction were performed using Advanced Normalization Tools (ANTs). Following a rigid-body motion correction, a temporal mean image was generated for time series of VASO and BOLD contrasts and their corresponding reversed phase-encoding data separately. Afterward, the mean image of the reversed phase-encoding data was rigidly aligned to the motion-corrected mean image of each contrast’s time series. The two images were subsequently fed to antsMultivariateTemplateConstruction2 (Avants et al., 2011) to perform a “meet-in-the-middle” registration using ANTs symmetric normalization (SyN) algorithm to obtain distortion-corrected mean images for each contrast. Next, all transformation and distortion-corrected warps were concatenated and applied using antsApplyTransforms with Lanczos interpolation. Note that distortion correction was applied in five out of the six datasets as opposite phase-encoding data were not available in one participant (see Section 2.3.2). The preprocessed VASO data were further corrected for BOLD contamination using the LN_BOCO program in LayNii (Huber et al., 2021). Following preprocessing, the BOLD-based fMRI data were aligned to the T1 image using the AFNI script “align_epi_anat.py”. The mean VASO image was first meticulously aligned to the T1-weighted image using ITK-SNAP (Yushkevich et al., 2006), utilizing its capability of allowing careful manual interventions to ensure high-quality alignment in the presence of EPI artifacts in some parts of the FOV that can confuse fully automatic registration approaches. Manual co-registration was guided by HC anatomical landmarks, with particular attention to alignment along the hippocampal long axis. The resulting transformation matrix was then applied to all VASO time series with fifth order B-spline interpolation. Further, to quantify physiological noise in the T1-aligned VASO and BOLD time series, anatomical Component Correction (aCompCor) was applied (Behzadi et al., 2007; Mascali et al., 2021). Specifically, 5 principal components were extracted separately from WM and CSF masks, resulting in 10 regressors in total. Prior to aCompCor implementation, the whole-brain WM and CSF masks were cropped to match the co-registered functional data and eroded using the FSL tool “fslmaths” with a Gaussian kernel of σ = 0.8 mm to ensure that no HC voxels were included in the anatomical masks. All stages of preprocessing were subject to careful visual inspection for quality control. Moreover, tSNR maps were computed in all functional time series to quantitatively assess the data quality.

#### 2.4.3 Extraction of laminar profiles in HC subfields

In line with previous studies (Ahmadi et al., 2024; Pfaffenrot et al., 2025), the co-registered VASO and BOLD time series were sampled as a function of depth using a custom MATLAB code. In short, GM signal in each HC subfield was sampled into 20 equidistant bins spanning the vertices of the HC inner and outer surfaces, which were generated by HippUnfold (DeKraker et al., 2022), with the signal in each bin averaged across 7262 vertices. To account for the pronounced curvature of the HC, the mid-thickness depth, also computed via HippUnfold representing the center of GM in the HC, was designated as a reference landmark for the sampling algorithm. In subiculum, CA1, and CA2, the sampling was further extended by an additional 10 bins beyond the inner surface to encompass the SRLM. The sampling was not applied to DG/CA4 due to the absence of a well-defined inner/outer surface boundary in these areas (see Supplementary Fig. S1).

#### 2.4.4 General linear model (GLM) analysis

GLM analysis was performed using Statistical Parametric Mapping (SPM12; Wellcome Trust Centre for Neuroimaging, London, UK) to separately estimate the VASO and BOLD activation maps for each stimulus condition. No spatial smoothing was applied. The fMRI data were high-pass filtered using a discrete cosine transform (DCT) with a cutoff period of 128 seconds (SPM default) to remove low-frequency drifts and subsequently convolved with a canonical hemodynamic response function. A design matrix was created, incorporating 2 regressors of interest, that is, autobiographical memory and math conditions, as well as 16 nuisance regressors including 6 motion estimates and 10 principal components derived from aCompCor. For each participant, a primary contrast of interest was calculated as the difference between beta-estimates of memory and math conditions. Statistical maps were computed across the entire slab volume of the fMRI data. To mitigate the problem of multiple comparisons with the increased number of voxels at high spatial resolutions, and enhance anatomical specificity, a GM mask cropped to the functional slab coverage was applied during cluster-level inference. For group-level analysis, a study-specific anatomical template with an isotropic spatial resolution of 0.75 mm^3^ was generated from the denoised T1-weighted images of all participants using the ANTs multivariate template construction algorithm. The statistical maps from first-level GLM analysis were subsequently co-registered to this template using the transformation matrices obtained during template construction.

The GLM analysis was also conducted on depth-dependent VASO and BOLD responses across HC subfields. Following the approach outlined by Pfaffenrot et al. (2025), the laminar time series were first z-transformed using the mean and standard deviations computed from the volumes corresponding to math condition.

## 3 Results

### 3.1 Part 1: Developing an HC-tailored VASO protocol

A schematic illustration of the optimized protocol is shown in Figure 1C and a detailed list of the protocol parameters is available in the following GitHub repository: https://github.com/layerfMRI/Sequence_Github/tree/master/Hippocampus.

#### 3.1.1 Mitigating methodological and physiological challenges of VASO in HC

**Fig. 2.**
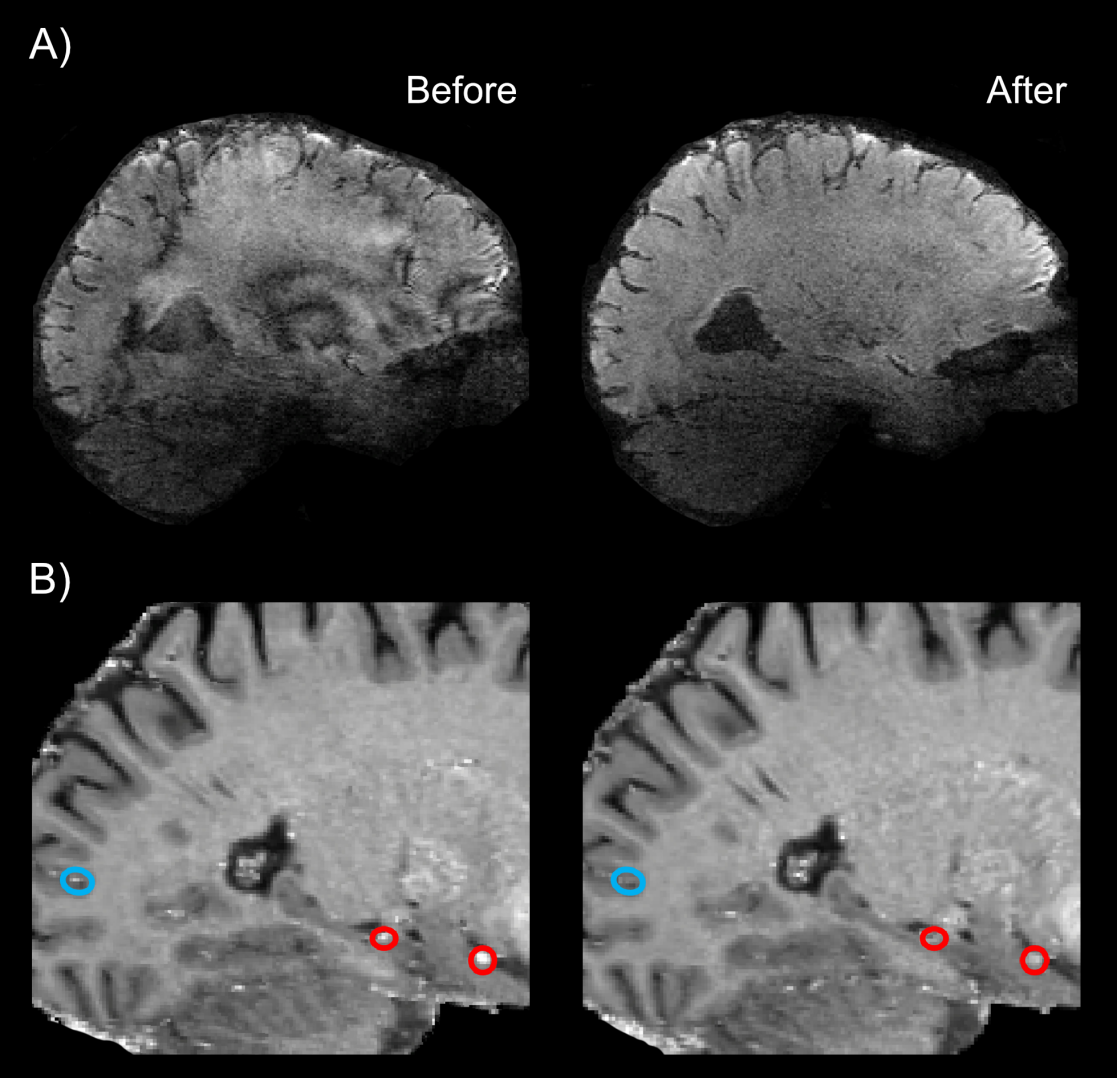
Characterization and mitigation of gradient-related and inflow artifacts in VASO-based laminar fMRI in HC. (A) Bandwidth adjustments, disconnecting third-order shim and read-out along the z-axis collectively mitigated the EPI phase errors*.* (B) Inflow effects were assessed using a series of adiabatic RF pulses of varying power. Blue and red circles indicate manually defined ROIs to assess inflow-related signal modulations in a posterior neocortical region (blue), the HC (middle red circle), and a more anterior temporal region (right red circle). The bottom right panel demonstrates that increasing the RF power of the inversion pulse decreases inflow-related signal modulations in large vessels outside of GM. This suggests that inflow effects are not expected in downstream microvessels of the GM tissue. EPI = echo planar imaging, ROI = regions of interest, HC = hippocampus, GM = gray matter.

**Fig. 3.**
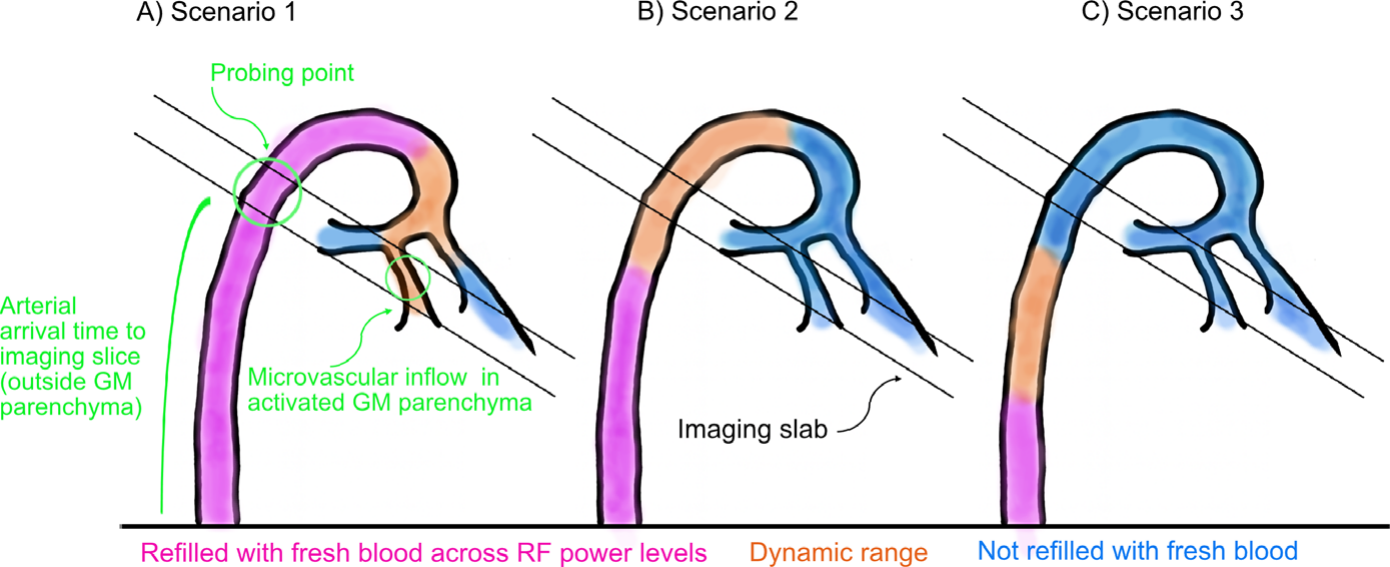
Schematic illustration of three envisioned inflow scenarios. Arterial arrival time occurs outside the gray matter (GM) parenchyma, marking the upstream probing point where inflow effects are measured. (A) In scenario 1, inflow of fresh blood extends beyond macro-vessels regardless of applied RF power level, likely compromising data quality. (B) In scenario 2, fresh blood inflow is restricted to large vessels with minimal effect on microvasculature. The dynamic range responsible for inflow effects becomes refilled only under weak inversion power and only during activity, but not under stronger inversion or during rest. Consequently, bright inflow-related spots decrease with increasing RF power (see Fig. 2B). (C) Fresh blood does not reach macro-vessels during the inversion time in scenario 3, indicating no contamination.

EPI quality was improved through (i) read out along the z-axis to minimize short-term eddy currents, (ii) bandwidth adjustments, and (iii) disconnecting third-order shim to decrease inductive coupling (Fig. 2A; see also Huber, Stirnberg, et al., 2025). To evaluate the inversion efficiency of inflowing blood in the HC and assess potential inflow contamination, a series of adiabatic RF pulses with varying power scales were applied. The aim was to determine whether inflow effects remain confined to upstream vascular compartments or propagate downstream into the HC microvasculature. It should be noted that the arterial arrival time, used to define the probing point, is measured in vessels located outside the GM parenchyma, serving as an upstream reference where neural activation effects are not present. Three potential scenarios were considered. (i) If fresh blood inflow extends beyond macro-vessels, inflow-related signal would remain high across RF power levels, indicating that inversion inefficiency affects smaller vessels downstream, including the HC microvasculature. (ii) If inflow is restricted to large upstream vessels, the inflow-related signal change should decrease with stronger RF power, suggesting minimal impact on the HC microvasculature. (iii) Finally, if no fresh blood reaches the macrovasculature during the inversion time, inflow-related signal would be absent across RF power levels, implying no inflow contamination (see Fig. 3). The results in Figure 2B support the second scenario. Inflow-related signal increased at lower RF powers and was attenuated with stronger inversion (optimized RF power scale = 3), indicating that inflow effects are mostly limited to larger vessels. Quantitative analysis of mean VASO signal intensities from three manually defined regions of interest (ROIs), a posterior neocortical region (blue circle in Fig. 2B), the HC (middle red circle), and a more anterior temporal region (right red circle) confirmed a marked decrease in signal intensity with increasing RF power: from 0.85 to 0.39, 1.001 to 0.66, and 0.94 to 0.73, respectively. Residual inflow signal in these vessels is not a concern, as they do not exhibit task-related functional changes and maintain constant flow between rest and task conditions. Upon optimizing inversion pulse parameters, no major inflow-compromised VASO signal was detected in HC. Notably, inflow modulations were comparable between the HC and posterior neocortical regions (Fig. 2B).

#### 3.1.2 Sequence stability across scanners and comparison of coil performance

**Fig. 4.**
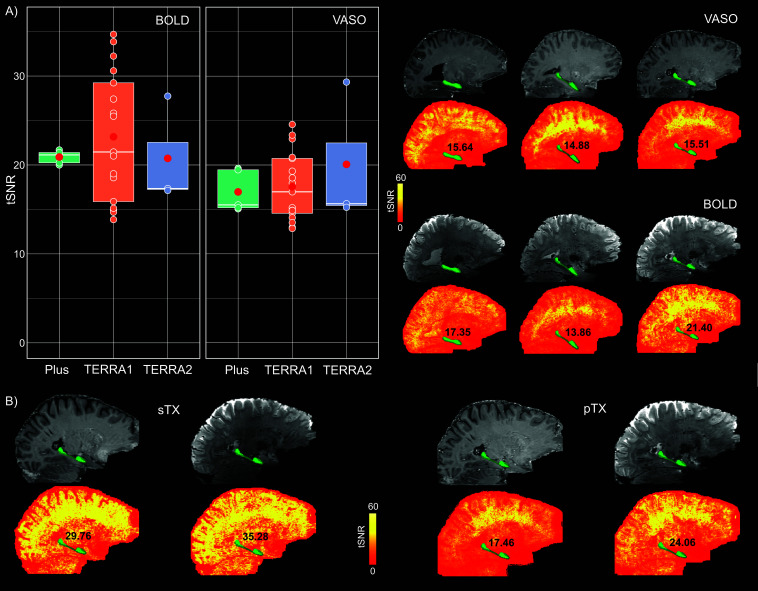
Consistency of tSNR in HC across scanners and head coils. (A) Mean HC-tSNR values for BOLD and VASO contrasts across two identical MAGNETOM TERRA and one classic 7T Plus scanners (N_TERRA1_ = 17, N_TERRA2_ = 3, N_Plus_ = 5). In the box plots, the white solid line denotes the median, the red circle represents the group mean, and the remaining scatter points reflect the individual data per scanner. The top right panel displays mean VASO and BOLD images along their corresponding tSNR maps in three randomly selected participants whose data are also shown in the box plots. Average HC-tSNR values are shown on the maps next to the anatomical location of HC, highlighted with a green mask. (B) Comparison of mean BOLD and VASO images together with their respective tSNR maps between sTX and pTX coils in the same participant, indicating higher values for the sTX coil. HC = hippocampus, tSNR = temporal signal-to-noise ratio.

Three conventional 7T scanners (see Section 2) were used in this study, allowing us to investigate the robustness of the HC-tailored VASO protocol across scanners. The mean tSNR in the HC and the mean image quality for both VASO and BOLD contrasts were comparable across different scanners, indicating that the performance of the optimized sequence is independent of scanner-specific factors (Fig. 4A). Due to the unequal number of data points per scanner, statistical testing was not conducted to assess between-group differences to avoid potential bias.

To investigate within-subject reliability of the optimized protocol, HC-tSNR values were compared across all runs in participants who underwent multiple scanning sessions. Across sessions, tSNR values were generally consistent for both BOLD and VASO, further supporting the stability of the optimized sequence (see Supplementary Table S3). Additionally, in one participant who was scanned using both sTX and pTX coils, HC-tSNR values were compared between coil types. As shown in Figure 4B, the sTX coil yielded slightly higher tSNR for both BOLD and VASO contrasts in this participant, suggesting a potential advantage for HC-focused laminar applications.

### 3.2 Part 2: Sequence validation with an autobiographical memory paradigm

#### 3.2.1 Disentangling brain activations between memory and math trials

**Fig. 5.**
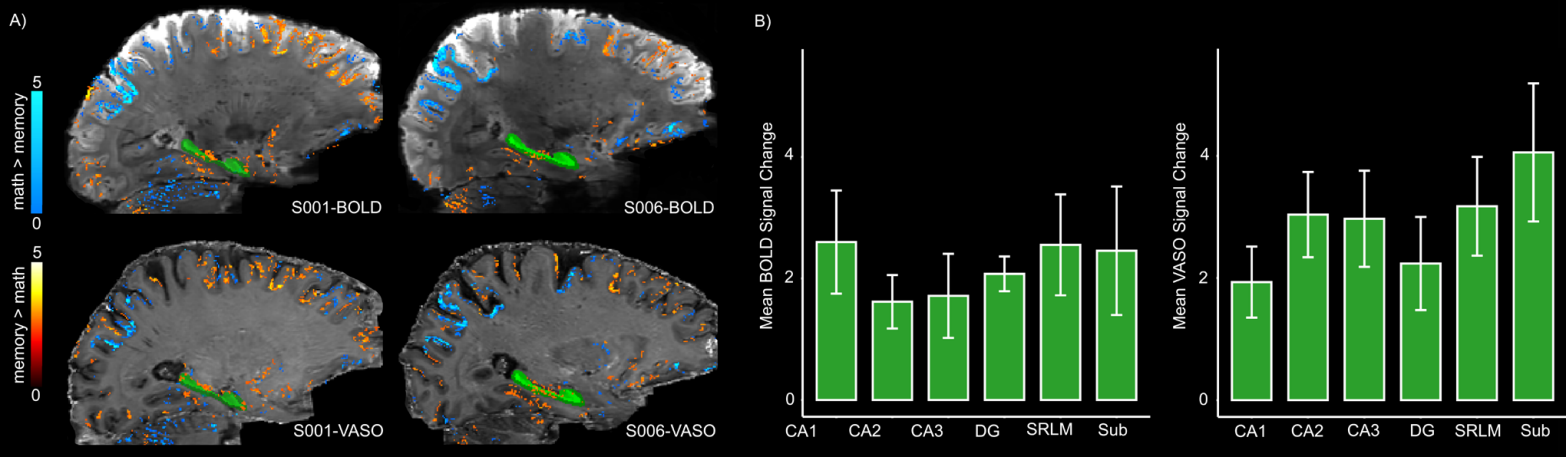
Brain activation maps and average signal changes across HC subfields for BOLD and VASO contrasts. (A) Representative activation patterns from two participants (S001 and S006) during memory and math trials overlaid on the mean BOLD (top) and VASO (bottom) images (p < 0.05, uncorrected). The green mask indicates HC. (B) Mean signal change for VASO and BOLD (left and right panels, respectively) across subfields derived from significant clusters within the HC for memory > math (as shown in panel A). The error bars indicate standard error of the mean (SEM). Note that here “DG” represents a merged ROI encompassing both the dentate gyrus and CA4.

First-level GLM analysis with a cluster extent threshold of 20 voxels revealed predominant activation in parietal regions during math operations, whereas memory retrieval elicited greater activity in the frontal cortex, parahippocampal cortex, and the HC in all participants. Importantly, the overall activity patterns were consistent between VASO and BOLD contrasts (see Fig. 5A).

As a descriptive summary of HC activation, the mean value and percentage of surviving voxels per subfield for both BOLD and VASO are listed in Supplementary Table S4. To examine whether hippocampal subfields contribute differentially or uniformly to autobiographical memory retrieval, signal change (quantified as the mean contrast estimate [β_memory_ − β_math_]) was extracted from conjunction ROIs (significant voxels for BOLD + VASO) within each subfield. As shown in Figure 5B, no significant differences were detected between subfields for either imaging contrast (VASO: χ^2^ (5) = 8.47, p = 0.13, BOLD: χ^2^ (5) = 8.09, p = 0.15). Given the small sample size, the non-parametric Friedman test was used instead of parametric alternatives such as repeated-measures ANOVA. To ensure that the observed findings were not influenced by potential circularity, an additional control analysis was conducted in which the mean signal change was extracted from the entire anatomically defined subfields, irrespective of activation (Supplementary Fig. S2). Similarly, this analysis revealed no significant differences between subfields (VASO: χ^2^ (5) = 2.85, p = 0.72, BOLD: χ^2^ (5) = 8.76, p = 0.11).

To assess the trade-off in sensitivity between BOLD and VASO, we quantified the average functional contrast-to-noise ratio (CNR) for memory > math contrast within the HC. In each participant, CNR was computed separately for BOLD and VASO as the absolute mean contrast estimate | βmemory – βmath | within the HC, divided by the square root of the mean residual variance derived from the GLM analysis in SPM, that is, “ResMS.nii”. Across participants, the median CNR in HC was 0.29 for BOLD and 0.25 for VASO (see also Supplementary Fig. S3). A paired Wilcoxon signed-rank test revealed no statistically significant difference between the two imaging contrasts in CNR (V = 19, p = 0.093).

Given the small number of participants (N = 6), we further performed sensitivity analysis by extracting the mean BOLD and VASO signal change for the memory > math contrast in each participant within two ROIs: a gray matter mask cropped to the functional slab coverage and the whole HC, that is, a composite ROI of all subfields. The standard deviation across participants was then computed for each ROI and imaging contrast. The resulting minimum detectable effect sizes (MDES) are reported in Supplementary Table S5, ranging from 0.144 to 0.55. For reference, the corresponding standardized MDES (one-sample Cohen’s *d*) for 80% power at α = 0.05 is 1.43, suggesting that statistical power is limited for detecting small effects at the group level.

Similar activation maps (nifti data available on Zenodo) were observed at the group level although the effects were less pronounced in the VASO dataset. Nevertheless, the similarity of the overall observed activation patterns during memory retrieval and math operations to previously reported activation profiles (Dehaene et al., 2005; Leelaarporn et al., 2024; McCormick et al., 2015; Rickard et al., 2000).

#### 3.2.2 Laminar profiles of HC subfields for memory vs math trials

**Fig. 6.**
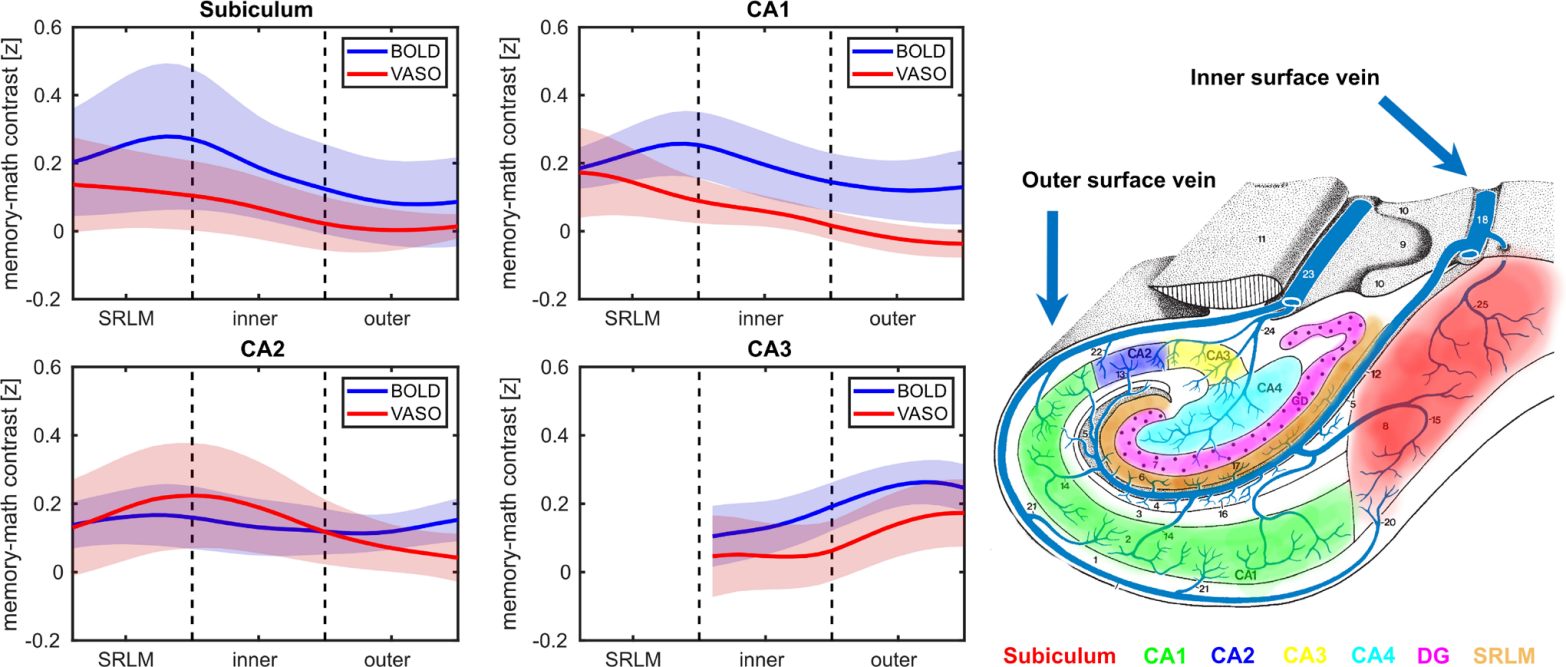
Laminar profiles of subfields for memory vs math contrast along with HC vascular architecture. In subiculum and CA1, the BOLD signal profiles (blue) peak at the SRLM near the inner surface, whereas in CA2, the BOLD increase is confined to the outer surface, likely reflecting subfield-specific draining veins’ bias. Conversely, the VASO signal (red) shows the opposite pattern in those subfields at the same depth. Shaded areas indicate SEM. Note that the laminar profiles are not estimated in DG and CA4 due to lack of a clear boundary between the inner and outer surfaces. The right panel displays a schematic illustration of HC venous supply, adapted from Duvernoy (2005).

Figure 6 illustrates the average depth-dependent profiles of HC subfields across all participants for the z-transformed memory > math contrast (see Section 2 and Pfaffenrot et al., 2025) in BOLD and VASO data. In subiculum and CA1, the laminar BOLD signal exhibited a peak at the SRLM adjacent to the inner surface, corresponding to superficial layers, followed by a decreasing pattern toward the outer surface. In CA2, the BOLD signal showed a modest elevation at SRLM, a dip in the inner surface and a subsequent increase at the outer surface. In contrast, CA3 displayed a largely monotonic increase in the BOLD signal across depths. Crucially, the increase in BOLD-driven laminar profiles across HC subfields aligns with the expected draining venous bias based on neuroanatomical evidence and a recent BOLD-based laminar fMRI study (Duvernoy, 2005; Pfaffenrot et al., 2025), that is, a more pronounced effect toward the SRLM/inner surface in the subiculum and CA1 and at the outer surface in CA2 (see Fig. 6). Interestingly, the VASO signal demonstrated a primarily inverse laminar trend relative to BOLD, manifesting a decreasing pattern at the SRLM/inner surface of subiculum and CA1 as well as the outer surface of CA2. To quantitatively substantiate this observation, we fitted a linear mixed-effect model (implemented via “nlme” package in R) in the subiculum, CA1 and CA2, showing visually divergent patterns. Specifically, the signal change was modeled as a function of depth, imaging contrast (BOLD vs VASO), and their interaction with random intercepts for participants and an AR(1) autocorrelation term to account for residual dependencies across adjacent depth bins.

In subiculum, the imaging contrast x depth interaction was not significant (β = -0.00022, p = 0.9), indicating that despite visually opposing patterns, the depth-dependent profiles of BOLD and VASO did not statistically differ in this subfield. However, there was a significant main effect of depth (β = -0.004, p = 0.0035), reflecting a decrease in signal magnitude with increasing depth for both BOLD and VASO. In CA1, the imaging contrast x depth interaction was highly significant (β = -0.005, p = 0.0005), demonstrating that the depth-dependent slope for VASO differed markedly from that of BOLD. Similarly, in CA2, the interaction effect was significant (β = -0.0035, p = 0.029), confirming opposite depth-dependent slopes between BOLD and VASO. In CA3, however, the laminar profiles of BOLD and VASO were comparable. Similar laminar profiles were also observed for non-transformed memory > math contrast (Supplementary Fig. S4).

#### 3.2.3 Voxel-wise and depth-dependent tSNR across HC subfields

**Fig. 7.**
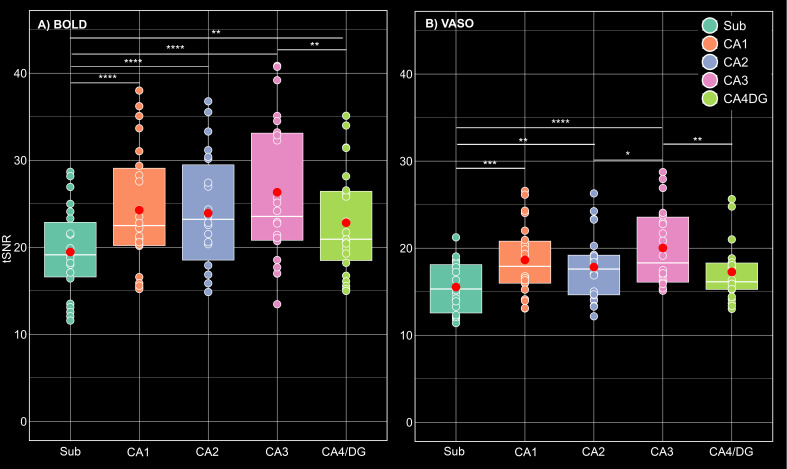
tSNR comparison across HC subfields for (A) BOLD and (B) VASO data. In the box plots, the white solid line indicates the median, the red circle represents the mean, and the remaining scatter points correspond to single runs. Note that tSNR values were computed throughout the entire acquisition period (150 volumes per run). Statistical significance is denoted by asterisks (*p < 0.05, **p < 0.01, ***p < 0.001, ****p < 0.0001).

To quantify signal stability across subfields, tSNR was computed at voxel level and as a function of depth. As depicted in Figure 7, the average voxel-wise tSNR for BOLD was slightly higher than VASO across all subfields. To further examine subfield-specific differences in tSNR, linear mixed-effect models were separately applied for BOLD and VASO data with subfields as fixed effects and participants as random intercept. For both contrasts, the models revealed a significant effect of subfield on tSNR. Tukey-corrected post hoc comparisons indicated that, for BOLD, all subfields exhibited significantly higher tSNR than the subiculum (all p < 0.01).

In VASO, tSNR in the subiculum was significantly lower than CA1, CA2, and CA3, but not CA4/DG (all p < 0.01). Moreover, across both contrasts, CA3 showed the highest tSNR. In BOLD, CA3 showed significantly greater tSNR than CA4/DG (p = 0.002). In VASO data, there was an additional significant difference between CA3 and CA2 (p = 0.019). All other pairwise comparisons did not reach statistical significance.

**Fig. 8.**
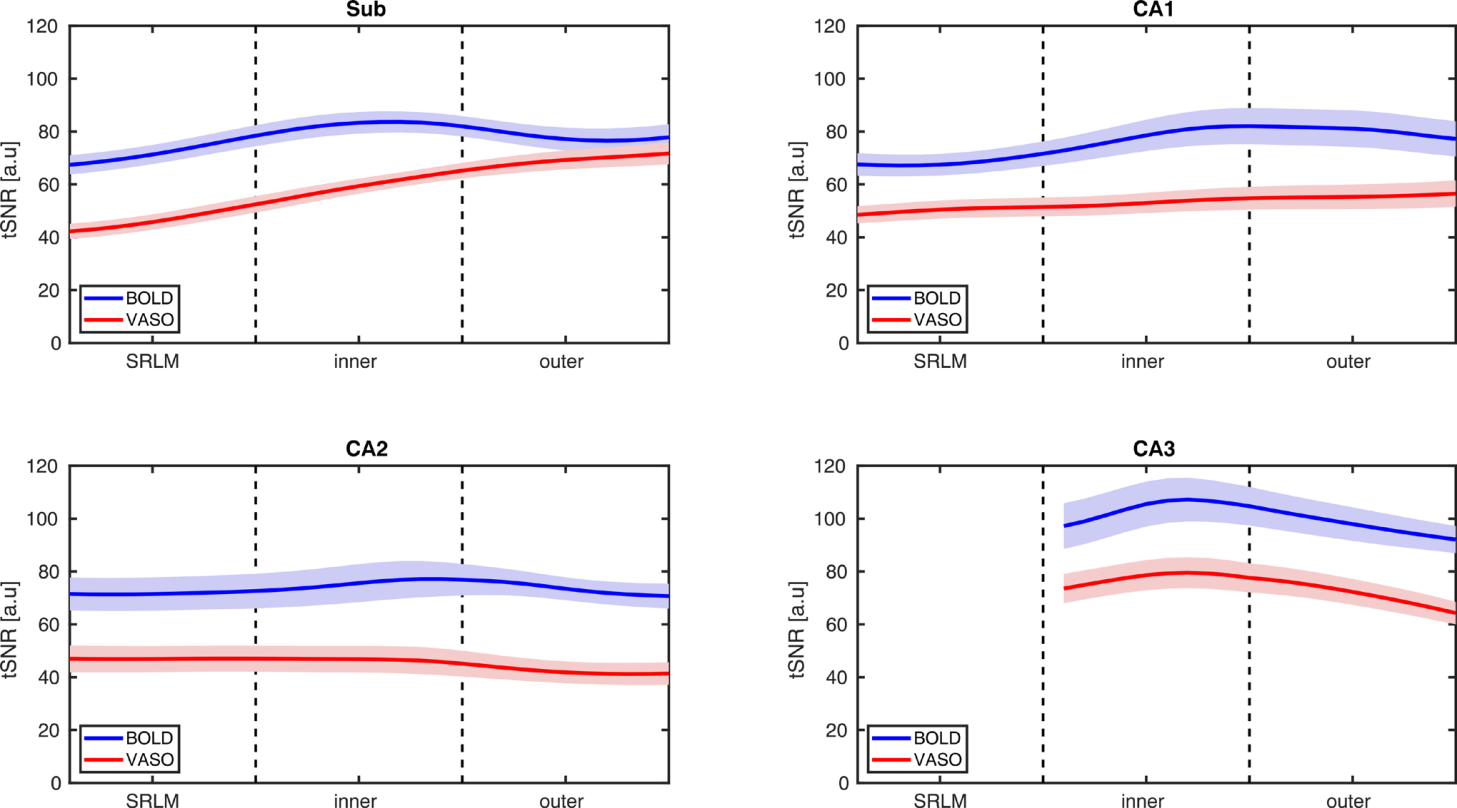
tSNR changes across subfields’ depths for both BOLD and VASO. Although tSNR is higher for BOLD than for VASO, its laminar profiles are similar across contrasts and remain largely stable as a function of depth in all subfields. The subiculum is the only exception, showing an increase from the SRLM toward the inner surface and a subsequent flattening at the outer surface. tSNR values were computed throughout the entire acquisition period (150 volumes per run).

Similarly, laminar profiles of tSNR were consistently larger for BOLD than for VASO and remained relatively constant across subfields’ depths, with the exception of the subiculum displaying an increasing trend in tSNR from SRLM toward the inner surface (Fig. 8). Notably, the laminar tSNR values were markedly higher than voxel-level values largely due to averaging over 7262 vertices in each depth bin, thereby suppressing random thermal noise by canceling uncorrelated fluctuations (see Section 2).

## 4 Discussion

Despite significant progress in harnessing laminar fMRI to uncover the directionality of information flow in the neocortical areas (Fracasso et al., 2018; Kok et al., 2016; Olman et al., 2012; Sharoh et al., 2019; van Dijk et al., 2021), analogous investigations in non-neocortical regions and particularly the HC remain limited (Ahmadi et al., 2024; Maass et al., 2014; Pfaffenrot et al., 2025). To date, the available studies have examined depth-dependent activity across HC subfields using GE-BOLD contrast whose signal is weighted toward large venous vessels. To mitigate the macrovasculature bias, we aimed to develop an HC-tailored VASO protocol. Our findings provide evidence for successful implementation of mesoscale VASO in human HC by (i) yielding sufficient signal quality with minimal artifacts across conventional 7T scanners and (ii) enabling reliable measurement of laminar-specific signal modulations across subfields during an autobiographical memory task.

### 4.1 VASO optimization in HC

The major innovation of this study lies in the acquisition strategies designed to address the methodological and physiological challenges of applying submillimeter VASO in the HC on conventional 7T scanners. The anatomical location of the HC renders it susceptible to B_0_ and B_1_ inhomogeneities, leading to more pronounced EPI artifacts and a subsequent decrease in SNR (Koster et al., 2018; Maass et al., 2014; Pfaffenrot et al., 2025). Additionally, similar to other inferior brain areas, the HC is located far from RF coil elements and often requires a relatively large matrix size and short T2* values for acquisition (Huber, Stirnberg, et al., 2025), factors that further exacerbate the SNR loss. We used pTX system and optimized the EPI protocol to boost the tSNR by decreasing FOV, GRAPPA, base resolution, and partial Fourier while increasing the bandwidth, and made further adjustments to other sequence parameters (see Section 2, and Fig. 1B&C). A prominent source of artifact in laminar fMRI is the appearance of Fuzzy ripples represented by low spatial frequency signal shadings (Huber, Stirnberg, et al., 2025). These artifacts are particularly severe in deep brain areas including the HC and are largely associated with eddy currents in ramp-sampling EPI. In SIEMENS whole-body scanners, these eddy currents are predominantly induced by inductive coupling with the third-order shim (Boulant et al., 2024). To mitigate Fuzzy ripples and improve the EPI equality, we unplugged third-order shim prior to data acquisition, reoriented the EPI read-out along the z-axis, and adjusted the bandwidth (Fig. 2A). Inflow-compromised VASO signals were largely mitigated using adiabatic RF pulses optimized for inversion efficiency (see Figs. 1C and 2B). After adjusting RF power scale, inflow effects in HC were similarly modulated to those observed in posterior neocortical regions, suggesting that the effects are primarily driven by larger arterial vessels with minimal impact on the HC microvasculature. In this study, TI1 was set to 1491 ms. Although arterial arrival to the HC via the posterior cortical and anterior choroidal arteries is shorter than in cortical gray matter, our inflow-characterization experiment confirmed that residual inflow effects remained largely confined to the macrovascular compartment (Figs. 2B & 3), thereby not compromising the VASO signal in the HC. Previous VASO studies have used TI values between 1100 ms (Finn et al., 2019; see Huber et al., 2017) and approximately 1500 ms (Huber et al., 2014), corresponding to the expected blood nulling time for typical hematocrit levels across sex and oxygenation state. Importantly, the experimentally utilized TI is generally a compromise between multiple factors: (i) Desired large coverage requires longer acquisition periods, that is, higher numbers of slices typically require longer TIs. (ii) Conversely, inflow contaminations in regions with shorter arterial arrival time necessitate shorter TIs. However, the effective blood nulling time also depends critically on inversion pulse performance, that is, inversion efficiency. In our earlier studies (Finn et al., 2019; Huber et al., 2017) performed on older scanner software versions (e.g. SIEMENS VB17A-UHF), SAR constraints restricted the inversion efficiency of large bandwidth inversion pulses. This made it necessary to decrease the TI. In contrast, newer generations of scanners allow inversion pulses with larger power and thus higher inversion efficiency enabling use a longer TI in the current study.

The optimized protocol showed strong reproducibility across three conventional SIEMENS 7T scanners (two identical TERRA and a classic 7T Plus), representing the most widely distributed 7T platforms globally, thereby underscoring its generalizability and practical utility. The comparison of head coils revealed a modest SNR advantage in HC for both BOLD and VASO contrasts with the sTX coil (Fig. 4B), which is consistent with reports on increased noise coupling of the lower row of receivers in the pTx coil (Huber, Morgan, et al., 2025). While the number of available datasets obtained with both coil types (N = 1) precludes any conclusion, the observed difference may reflect g-factor amplification in the pTX coil (Gosselink et al., 2021). Future studies are, thus, warranted to explore this effect systematically in a larger cohort.

### 4.2 Functional validation using a memory task

To assess the sensitivity of the optimized sequence, we employed an autobiographical memory paradigm. In line with previous studies (Leelaarporn et al., 2024; McCormick et al., 2015; Pfaffenrot et al., 2025), we observed HC, parahippocampal, and frontal activity during memory retrieval for both BOLD and VASO data. Notably, both imaging contrasts yielded consistent activation patterns (Fig. 5A). Further investigation of BOLD and VASO signal change in significant clusters within the HC and across the entire anatomically defined subfields showed no differential activation across subfields (Fig. 5B, & Supplementary Fig. S2), suggesting a coordinated engagement of HC during autobiographical memory retrieval. This is partially in agreement with the findings of a recent study (Leelaarporn et al., 2024) reporting no significant difference in the activation of CA1 to CA3, DG/CA4, and the subiculum. However, that study employed manual segmentation of the subfields and included pre/parasubiculum that showed higher signal change than all other subfields. In contrast, we used HippUnfold (DeKraker et al., 2022) to automatically segment the subfields that does not delineate the pre/parasubiculum area. This may account for the absence of a distinct subfield effect in our results. Collectively, the convergence of the observed activation maps between BOLD and VASO coupled with their correspondence to regions identified in prior studies (Dehaene et al., 2005; Leelaarporn et al., 2024; McCormick et al., 2015; Rickard et al., 2000) supports the robustness of the optimized sequence in detecting task-related activity in the HC.

Laminar analyses revealed distinct activation profiles in each subfield. Specifically, the laminar BOLD responses in CA1 showed a peak at the transition zone between SRLM and the inner surface, while in CA2 a modest increase in BOLD signal was observed toward the outer surface. These findings are largely consistent with a recent BOLD-based laminar fMRI study (Pfaffenrot et al., 2025) and the neuroanatomical evidence on hippocampal venous supply (Duvernoy, 2005), suggesting a potential contribution of draining vessels to the observed BOLD signal. In contrast, VASO signal profiles exhibited an inverse trend at the SRLM/inner surface border of CA1 as well as the outer surface of CA2, likely reflecting its sensitivity to microvascular CBV changes. A similar diverging trend between BOLD and VASO was apparent in subiculum at SRLM/inner surface border, although this difference did not reach statistical significance. In CA3, the laminar profiles of BOLD and VASO were comparable, both showing a peak at the outer surface (corresponding to deep layers). This could potentially demonstrate the contribution of trisynaptic pathway to autobiographical memory retrieval via pattern completion, a key computational function governed by CA3. This process involves the reactivation of an entire memory or episode from partial cues and is thought to rely on recurrent autoassociative connections in deep layers of CA3, likely at stratum radiatum and proximal apical dendrites (Le Duigou et al., 2014; Marr, 1971; Witter, 2007), toward its outer surface. Altogether, these findings indicate that the optimized VASO protocol can resolve depth-dependent activity in the HC.

It should be noted that the overall amplitude of VASO signal change was relatively comparable with BOLD at the voxel level (see Fig. 5B and Supplementary Fig. S2). Further, although the laminar BOLD responses were larger than VASO across all subfields (Fig. 6), the difference in response amplitude appears to be smaller than prior studies that have shown substantially smaller task-evoked VASO signal change in neocortical regions (Huber et al., 2017). The observed discrepancy is hypothesized to reflect true regional vascular characteristics and reduced sensitivity of VASO to physiological noise relative to BOLD, rather than methodological factors such as TR/TI choice, NORDIC denoising, inflow effects, or residual BOLD contamination. This interpretation is consistent with previous studies showing higher arterial blood volume (Donahue et al., 2010; Rane et al., 2016) in the HC than in the neocortex and a similarly reduced gap in depth-dependent response amplitudes between BOLD and VASO under different acquisition settings (Häkkinen et al., 2025).

tSNR comparisons at both the voxel level and across subfields’ depths revealed similar patterns between BOLD and VASO, further supporting the stability and robustness of the optimized sequence (Figs. 7 & 8), although tSNR values were higher for BOLD than for VASO at both scales. At the voxel level, tSNR differed significantly across subfields, with the subiculum showing lower tSNR and CA3 exhibiting the highest values, likely reflecting anatomical and susceptibility-related variation in signal quality for both contrasts rather than neural differences. Laminar profiles of tSNR were largely depth-invariant across all subfields except the subiculum where both BOLD and VASO showed a relatively monotonic increase from SRLM to the inner surface, plateauing at the outer surface. The close correspondence of laminar tSNR between BOLD and VASO further indicates the effectiveness of the optimized sequence in capturing reliable depth-resolved signal across HC subfields. It should be noted that the laminar profiles of tSNR were substantially larger than the average tSNR values at voxel level. This increase is primarily due to averaging the signal across 7262 vertices within each depth bin (see Section 2) prior to computing the laminar tSNR, reducing the contribution of random thermal noise. As such, the remaining variability in the depth-dependent tSNR is largely dominated by physiological noise and task-related fluctuations (relevant to detection sensitivity). In contrast, voxel-wise tSNR is directly calculated from individual voxels and averaged across the entire subfields ROIs that is presumably influenced by thermal noise (relevant to sequence quality assessment).

The present study has a few limitations that should be acknowledged. First, the observed gain in the tSNR for the optimized sagittal protocol comes along at the cost of missing the other hemisphere and the contralateral HC, thereby lowering the statistical power of fMRI response modulations. Second, the sample size was relatively small (N = 13). This is particularly relevant for part 2, the validation study, where the GLM results and the laminar profiles were derived from six datasets reducing the statistical power of the analyses. The observed effect sizes in Figures 5 and 6 were relatively small, which may be partially attributed to the limited amount of usable data. Although we acquired at least three functional runs per participant, some runs were discarded due to motion artifacts (see Supplementary Table S1). Moreover, each run contained 150 volumes after preprocessing, whereas in the previous BOLD-based laminar fMRI study (Pfaffenrot et al., 2025), 489 volumes were acquired per run, resulting in a larger magnitude of the depth-dependent signal. The concomitant acquisition of BOLD and VASO data in our protocol leads to a lower temporal resolution, limiting the number of volumes that could be acquired within a reasonable scan duration, as extending the acquisition time to collect more volumes would likely compromise data quality due to motion. Consistent with the small sample size, the sensitivity analysis demonstrated limited power to detect small effects. As such the observed modest effect sizes should be interpreted with caution, in line with the feasibility-oriented objective of this methodological study. Further, we applied NORDIC denoising (Knudsen et al., 2025; Vizioli et al., 2021) to boost the tSNR of the data. However, recent reports suggest that NORDIC may attenuate task-relevant signal variance (Faes et al., 2024), potentially contributing to the modest effect sizes observed in our study. To assess the potential impact of NORDIC denoising, we rerun the analysis in a randomly selected participant without applying NORDIC and compared the average tSNR values in HC and voxel-wise GLM results for memory and math trials (see Supplementary Table S6 and Supplementary Fig. S5). In the absence of NORDIC, both HC tSNR and surviving significant clusters were markedly decreased. As such, we opted to proceed with NORDIC-denoised data at the potential cost of response amplitudes (Faes et al., 2024). Nevertheless, it should be highlighted that the potential influence of NORIDC on response amplitudes warrants further investigation. Lastly, we used the default canonical HRF from SPM12, whereas prior work has shown that subcortical regions may exhibit distinct HRF nonlinearities compared with the neocortex (Lewis et al., 2018). To evaluate robustness of the observed activation patterns to HRF idiosyncrasies, we rerun voxel-wise GLM analysis using (i) the canonical HRF plus temporal derivatives and (ii) a finite impulse response (FIR) model in a representative participant. The activation maps for the memory > math contrast obtained with the canonical HRF with temporal derivatives were nearly identical to those modeled with the canonical HRF alone both for BOLD and VASO that might be due to the relatively long TR used here compared with the HRF time dynamics. Implementing an FIR model (with 9 seconds window length and 3 bins) yielded a similar activation pattern, albeit with reduced spatial extent (see Supplementary Fig. S6). This reduction is expected because the FIR model introduces more regressors per condition, decreasing statistical efficiency and thereby lowering sensitivity. Altogether, these additional analyses suggest that the observed activity maps are not driven by the assumptions about the shape of the HRF response.

The primary goal of this study was to evaluate the feasibility and validity of an HC-tailored VASO protocol, both of which are supported by our findings. Nevertheless, future studies with larger cohorts and optimized modeling of HRF will be necessary to achieve a higher statistical power. In conclusion, this study demonstrates that mesoscale VASO, when carefully optimized, is a viable tool for resolving laminar activation profiles in human HC. The protocol’s reproducibility across 7T platforms and sensitivity to task-evoked signals establish a strong foundation for future research to explore the depth-dependent properties of the HC subfields during complex cognitive functions and their alterations in neurodegenerative diseases and epilepsy. In the latter context, circuit-level alterations in HC, particularly in CA1, have been implicated in the heterogeneity of macroscale manifestations in temporal lobe epilepsy. EEG and conventional fMRI blur signal across parallel microcircuits, potentially masking deep vs superficial CA1 differences or subfield-specific patterns that might influence seizure onset, propagation, and clinical outcomes. By reducing draining-vein bias, mesoscale VASO may provide a promising avenue to disentangle these pathways in vivo and improve presurgical mapping and phenotyping (Aitken et al., 2025; Farrell et al., 2019).

## Supplementary Material

Supplementary Material

## Data Availability

Anonymized data have been deposited on Zenodo: https://zenodo.org/records/16032692. All original codes are provided in this GitLab repository: https://gitlab.ruhr-uni-bochum.de/neuropsy/vaso_hc.
