# Supplementary material for: Blood volume-sensitive laminar fMRI with VASO in human hippocampus: Capabilities and biophysical challenges at clinical 7T scanners
Source: Imaging Neurosci (Camb). 2026 Apr 9;4:IMAG.a.1197. doi: 10.1162/IMAG.a.1197 (PMC13069395; doi:10.1162/IMAG.a.1197)
Supplement: Supplementary Material [file IMAG.a.1197_supp.pdf]

Supplementary Materials

Participants' ID	Sessions	Acquired runs	Discarded runs due to motion artifacts	Total analyzed runs
**001**	3	12	3	9
**002**	1	3	2	1
**003**	1	3	1	2
**004**	1	4	1	3
**005**	1	3	0	3
**006**	2	7	3	4

Supplementary Table 1. Overview of the number of acquired sessions and functional runs from six participants whose data were analyzed for part 2 of the current study, focused on validating the HC-tailored VASO sequence using an autobiographical memory task.

Participant's ID	Run number	Contrast	Abs. motion		Rel. motion	
			max	mean	max	mean
001	1	BOLD	2.24	1.02	0.52	0.17
001	1	VASO	2.05	1.02	0.66	0.16
001	2	BOLD	1.7	0.94	0.69	0.21
001	2	VASO	1.83	1.03	0.65	0.23
001	3	BOLD	1.99	1.11	1.06	0.26
001	3	VASO	1.94	1.17	0.97	0.25

001	4	BOLD	1.57	0.95	0.74	0.25
001	4	VASO	1.53	0.98	0.62	0.23
001	5	BOLD	2.24	0.99	0.72	0.24
001	5	VASO	2.25	1.05	0.64	0.21
001	6	BOLD	2.01	0.96	0.59	0.22
001	6	VASO	2	0.91	0.79	0.2
001	7	BOLD	2.33	1.24	0.59	0.17
001	7	VASO	2.29	1.25	0.45	0.14
001	8	BOLD	1.89	0.92	0.52	0.18
001	8	VASO	1.79	0.88	0.47	0.17
001	9	BOLD	1.66	1.14	0.61	0.2
001	9	VASO	1.73	1.15	0.5	0.18
002	1	BOLD	2.11	1.16	2.09	0.25
002	1	VASO	2.12	1.1	2	0.2
003	1	BOLD	0.78	0.63	0.15	0.08
003	1	VASO	0.79	0.65	0.22	0.07
003	2	BOLD	1.89	0.99	0.49	0.13
003	2	VASO	1.84	0.96	0.38	0.12
004	1	BOLD	2.32	1.42	0.33	0.11
004	1	VASO	2.28	1.4	0.31	0.09
004	2	BOLD	2.48	1.68	0.67	0.21
004	2	VASO	2.48	1.65	0.56	0.2
004	3	BOLD	1.21	0.68	0.63	0.2
004	3	VASO	1.13	0.64	0.59	0.2
005	1	BOLD	2.44	1.44	0.59	0.2

005	1	VASO	2.36	1.2	0.44	0.2
005	2	BOLD	1.01	0.51	0.37	0.17
005	2	VASO	1.06	0.54	0.35	0.17
005	3	BOLD	0.86	0.49	0.32	0.16
005	3	VASO	0.86	0.47	0.29	0.16
006	1	BOLD	0.67	0.37	0.44	0.14
006	1	VASO	0.77	0.38	0.53	0.13
006	2	BOLD	0.56	0.24	0.46	0.14
006	2	VASO	0.5	0.31	0.4	0.14
006	3	BOLD	1.63	0.91	0.42	0.15
006	3	VASO	1.64	0.9	0.63	0.16
006	4	BOLD	0.79	0.43	0.49	0.1
006	1	BOLD	0.76	0.43	0.44	0.08

Supplementary Table 2. Summary of maximum and mean absolute and relative motion estimates for every participant and fMRI run. Absolute motion is defined as overall displacement compared to the reference volume (first volume) while relative motion estimates refer to displacement of each volume compared to the previous volume.

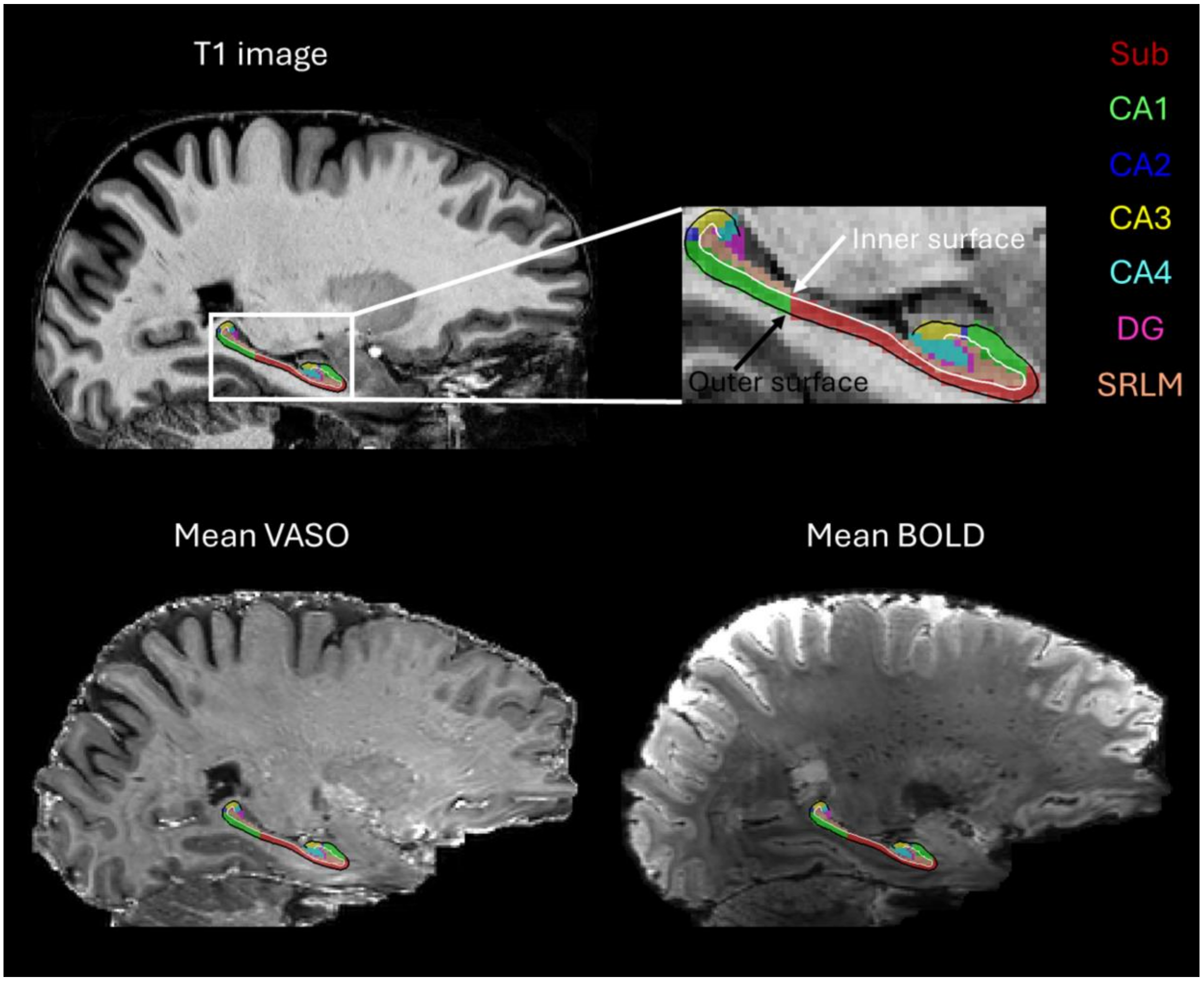

Supplementary Figure 1. Hippocampal subfield segmentation and surface boundary delineation overlaid on a T1-weighted image from one participant. The bottom panel is the same as above but displayed on co-registered mean VASO and BOLD images. Subfield labels and inner/outer surfaces were generated using HippUnfold. Sub = subiculum, CA1-CA4 = cornu ammonis areas 1 to 4. DG = dentate gyrus, SRLM = stratum radiatum lacunosom moleculare.

Participant's ID	Session	Run1	Run2	Run3	Run4	Contrast
**001**	1	15.88	14.93	14.69	15.13	BOLD
		14.07	12.98	12.86	13.52	VASO
	2	-	20.003	21.13	-	BOLD
		-	19.62	19.46	-	VASO
	3	-	21.69	21.40	20.24	BOLD
		-	15.07	15.51	15.19	VASO
**006**	1	-	-	25.82	25.48	BOLD
		-	-	19.30	18.55	VASO
	2	21.01	27.41	-	Not acquired	BOLD
		14.57	17.97	-		VASO

Supplementary Table 3. HC-tSNR values across all runs of the two participants who underwent multiple scanning sessions. Although 3 participants completed multiple sessions (see Methods), in the first session of one of these participants, only the reference 3D GE-EPI sequence with BOLD contrast was used while multiple readout parameters were adjusted. As such, no VASO data are available across sessions for this participant. For participant (001), four sessions were acquired, of which one was allocated to coil comparisons (see Figure 4.B). The tSNR values of all runs across the remaining sessions along with those of participant 006 are reported here. The consistency of the tSNR values across sessions demonstrates within-subject reliability of the optimized protocol. Dashed lines indicate discarded runs due to motion artefacts.

Number of Voxels	CA1	CA2	CA3	CA4/DG	Subiculum	SRLM
Entire anatomical ROI	2253 ± 82	340 ± 43	809 ± 38	746 ± 49	1229 ± 26	1449 ± 42
Surviving clusters (BOLD)	84 ± 33 (3.72%)	11 ± 4 (3.23%)	25 ± 9 (3.09%)	19 ± 5 (2.54%)	26 ± 13 (2.11%)	53 ± 17 (3.65)
Surviving clusters (VASO)	58 ± 42 (2.57%)	12 ± 4 (3.52%)	26 ± 13 (3.21%)	16 ± 10 (2.14%)	21 ± 9 (1.70%)	51 ± 35 (3.51%)

Supplementary Table 4. Number and percentage of voxels in each subfield. The values indicate the mean ± standard error of the mean. The first row shows the total number of voxels per anatomically defined subfields while the second and third rows show the number of surviving significant voxels for memory > math contrast for BOLD and VASO respectively.

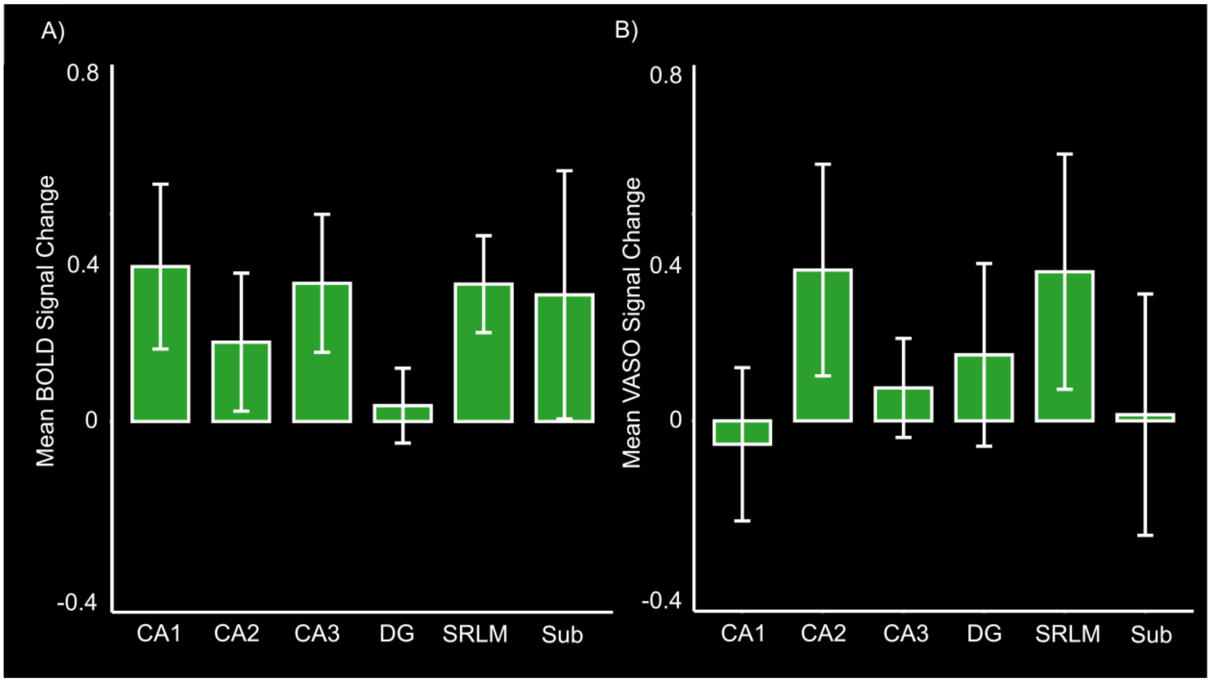

Supplementary Figure 2. Mean signal change for memory > math contrast across entire anatomically defined subfields with no statistical thresholding for **A)** BOLD and **B)** VASO. Overall response amplitudes are similar between BOLD and VASO. Note that voxel averaging across whole subfields may obscure depth-dependent effects in VASO as evident in subiculum and CA1 where contributions of laminar profiles with positive and negative responses in inner and outer surface (Figure 6) are cancelled when spatially pooled.

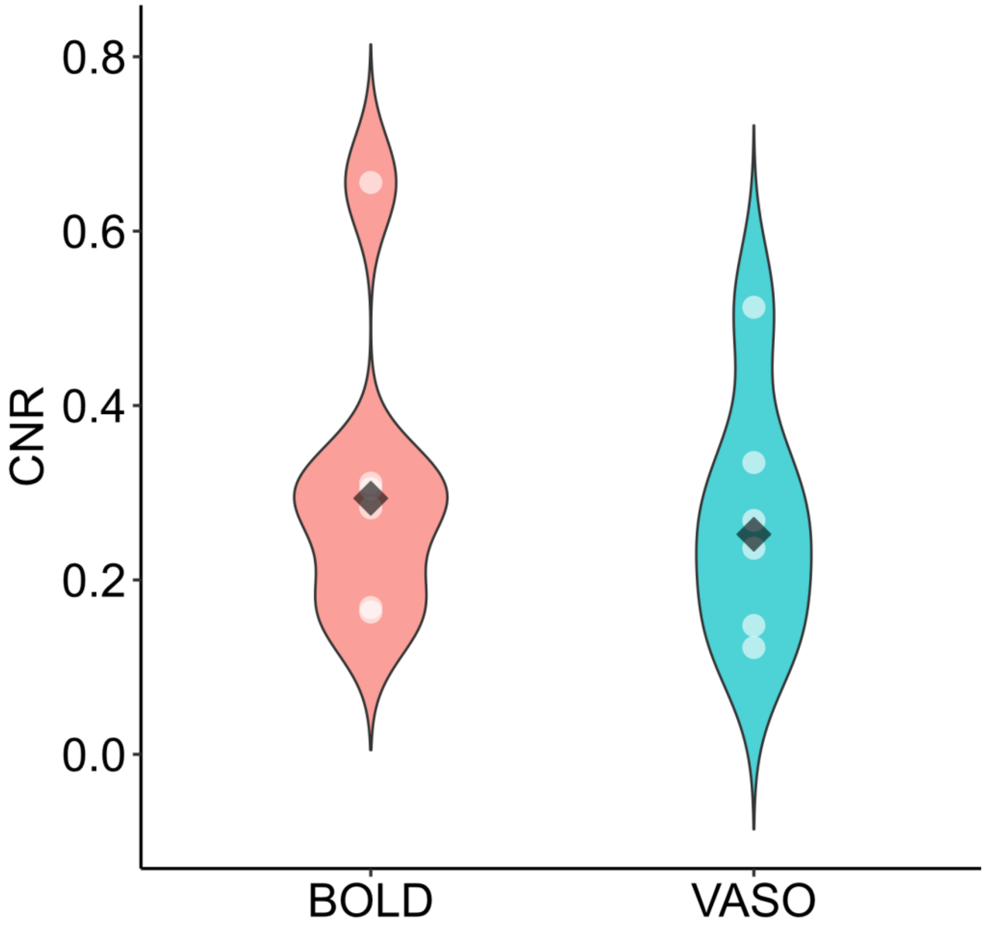

Supplementary Figure 3. Mean contrast-to-noise ratio (CNR) in the HC for the *memory* > *math* contrast. White dots represent the mean CNR value for each participant, and black diamonds indicate the group median for each imaging contrast (BOLD and VASO).

Imaging contrast	ROI	Mean	SD	MDES (raw contrast units)
BOLD	GM	0.07	0.19	0.27
VASO	GM	0.06	0.10	0.14
BOLD	HC	0.29	0.39	0.55
VASO	HC	0.10	0.33	0.47

Supplementary Table 5. Minimum detectable effect size (MDES) for memory > math contrast across imaging contrasts and ROIs. The MDES is reported in raw contrast units, calculated as d_{min} x SD where d_{min} is the standardized MDES (one-sample Cohen's *d*) for n = 6, 80% power and α = 0.05 that is equal to 1.43. Note that the value for d_{min} was obtained using R package 'pwr'.

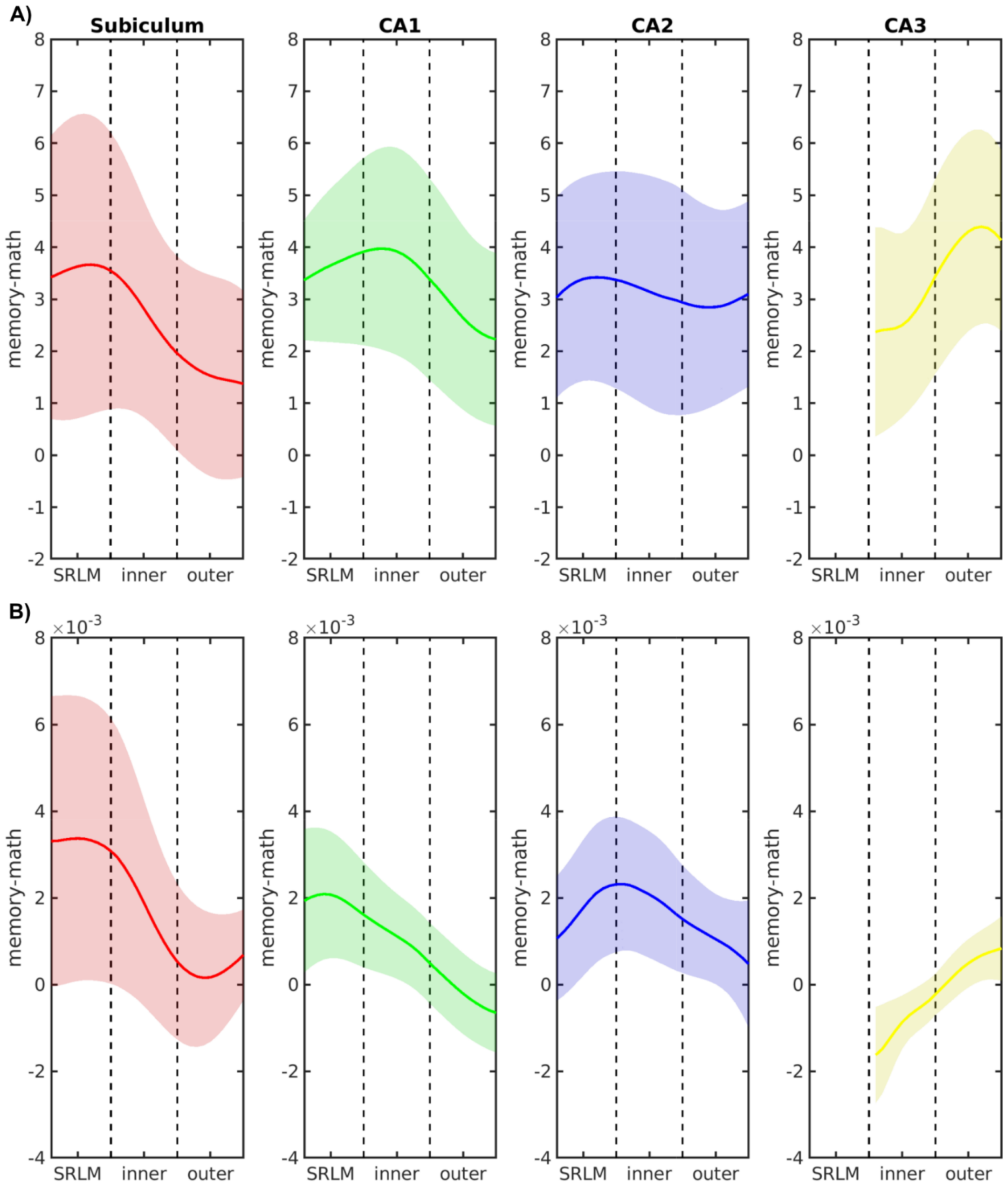

Supplementary Figure 4. Laminar profiles of HC subfields for memory vs math contrast (non-transformed). **A)** BOLD signal modulations across the depths of subfields **B)** VASO signal modulations. The laminar profiles of both BOLD and VASO are largely consistent with the patterns observed in z-transformed memory vs. math contrast (see Figure.6).

Run number	Imaging contrast	With-NORDIC<br>HC-tSNR	Without-NORDIC<br>HC-tSNR
2	BOLD	34.68	14.08
	VASO	23.26	6.25
3	BOLD	30.59	13.65
	VASO	20.86	6.33

Supplementary Table 6. Mean HC-tSNR values for BOLD and VASO contrasts with and without NORDIC denoising in a randomly selected participant (S003). Application of NORDIC results in substantial increase in tSNR for both imaging contrasts.

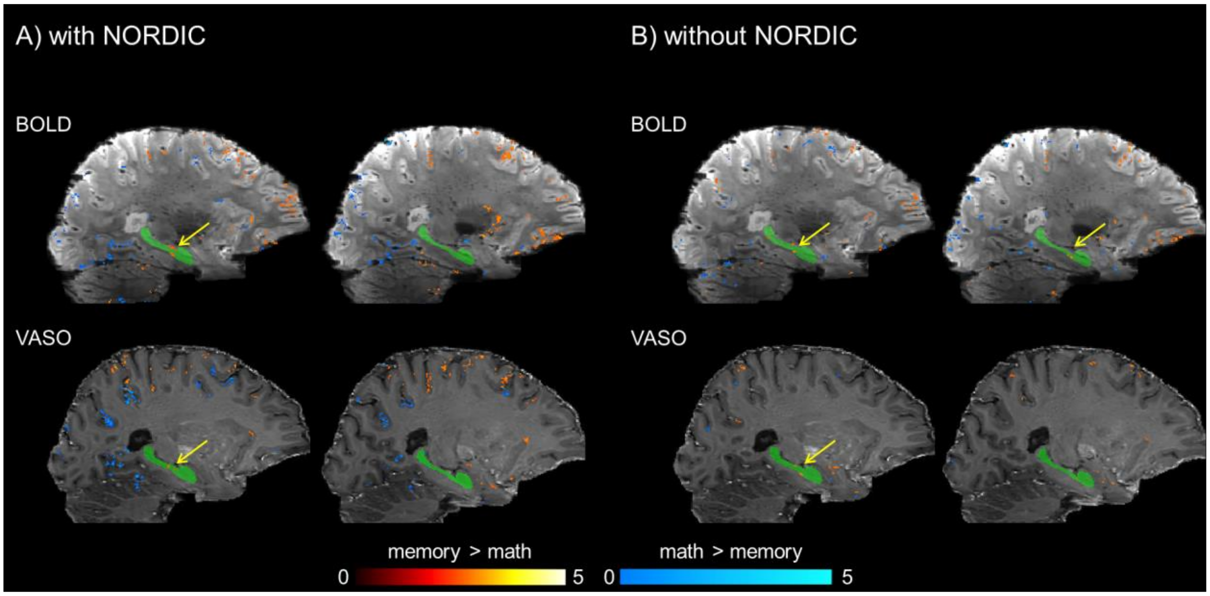

Supplementary Figure 5. Activation patterns during memory and math trials in a randomly selected participant (S003) overlaid on two slices of BOLD and VASO **A)** with NORDIC denoising and **B)** without application of NORDIC ($p < 0.05$ uncorrected). Although the overall activation is relatively small, likely due to the limited usable data (2 runs in this participant, see Supplementary Table 1), the surviving clusters are markedly decreased when NORDIC denoising is not applied.

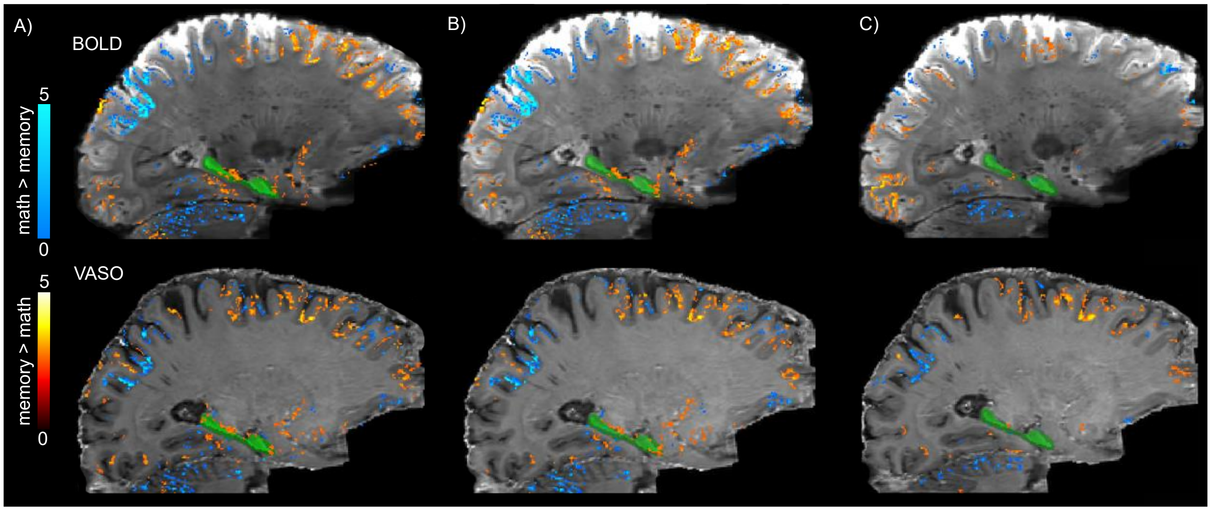

Supplementary Figure 6. Effect of different HRF response models on activity maps during memory and math trials ($p < 0.05$ uncorrected) in participant S001 for BOLD (top) and VASO (bottom). **A)** The activation patterns (identical to Figure. 5A) were based on GLM analysis with a canonical HRF alone. **B)** Activity maps were derived from canonical HRF with time derivatives. **C)** Significant activity clusters obtained using an FIR model. The overall activity patterns remain largely preserved across HRF models, suggesting that the observed activation does not strongly dependent on assumptions about the HRF shape.
